# Comorbidities in people living with HIV/AIDS and their impact on outpatient dental care

**DOI:** 10.1590/1807-3107bor-2025.vol39.035

**Published:** 2025-04-04

**Authors:** Maria Fernanda BARTHOLO, Jefferson Rocha TENÓRIO, Natália Silva ANDRADE, Cristiane Barbosa SILVEIRA, Karem López ORTEGA, Fabiana MARTINS, Marina GALLOTTINI

**Affiliations:** (a) Universidade de São Paulo – USP, Special Care Dentistry Center, Department of Stomatology, São Paulo, SP, Brazil.; (b) Universidade Federal do Rio de Janeiro – UFRJ, School of Dentistry Department of Pathology and Oral Diagnosis, Rio de Janeiro, RJ, Brazil.; (c) Universidade Federal do Sergipe – UFS, School of Dentistry, Department of Dentistry, São Cristóvão, SE, Brazil.

**Keywords:** HIV, HIV Long-Term Survivors, Acquired Immunodeficiency Syndrome

## Abstract

The objective of this study was to estimate the prevalence of comorbidities among people living with HIV/AIDS (PLWHIV) attending a dental outpatient clinic and discuss the impact of these comorbidities on dental management. A cross-sectional observational study evaluated 238 PLWHIV attending a specialized dental outpatient clinic in Brazil. We collected sociodemographic data, self-reported and physician-diagnosed comorbidities, hemogram results, CD4+ T cell count, viral load, use of combined antiretroviral therapy (cART), and information on harmful habits. The most prevalent comorbidities were sexually transmitted infections (STIs) (116/238; 48.7%), psychiatric disorders (105/238; 44.1%), and lipodystrophy (97/238; 40.8%). Men were more likely to have STIs (OR 4.0) and tuberculosis (OR: 2.5) (p < 0.05). Age ≥ 50 years increased the risk of diabetes mellitus by 2.6 times (p < 0.05). The risk of lipodystrophy (OR: 2.99, 95%CI 1.44–6.19) and psychiatric disorders (OR: 2.13, 95%CI 1.01–4.47) was greater in those who had been diagnosed with HIV for more than 20 years. In summary, psychiatric disorders and severe hematological alterations, such as anemia and neutropenia, are significant comorbidities that may limit dental treatment of HIV-positive patients. These findings underscore the need for integrated medical and dental care to address the complex health needs of PLWHIV.

## Introduction

Since the human immunodeficiency virus (HIV) and acquired immunodeficiency syndrome (AIDS) were first described in 1981, health professionals have witnessed significant changes in the general and oral health of people living with HIV (PLWHIV).^1^ With the advent of highly active antiretroviral therapy (HAART) in 1996, more recently referred to as combined antiretroviral therapy (cART), HIV infection became a manageable chronic condition. As a result, life expectancy has increased.^2^

However, the potential long-term impacts of antiretroviral medication, the prolonged effects of HIV-induced immune dysregulation and inflammation, and the persistence of behavioral risks (such as tobacco and other substance use) appear to exacerbate and accelerate the onset of age-related noncommunicable morbidities. Studies conducted at various centers have demonstrated that people living with HIV have high rates of atherosclerosis, diabetes, chronic kidney disease, and various malignancies.^3,5^

While it is challenging to determine whether HIV infection accelerates aging or merely increases the risk of chronic comorbidities, Siddiqi et al.^1^ demonstrated that the trend towards chronic comorbidities increases among older patients with HIV compared with to counterparts without HIV. Three of the eight major chronic illnesses (liver disease, kidney disease, and neurological disease) had a higher prevalence among hospitalized patients with HIV than among hospitalized patients without HIV, although the differences decreased over time.^1^

Given this scenario, the oral medicine specialty has seen significant changes in oral diseases and comorbidities in PLWHIV. In countries with widespread access to cART, as is the case in Brazil, advances in medical care for HIV infection have drastically reduced opportunistic infections and AIDS-defining malignancies. On the other hand, non-AIDS-defining cancers, non-AIDS-related comorbidities, and specific oral diseases such as periodontitis and salivary gland dysfunction have become more prevalent.^6-12^

Since the dental management of medically complex patients requires knowledge of their diseases or conditions, dentists should be aware of the medical conditions that PLWHIV may present to anticipate undesirable complications due to dental treatment. This study aimed to assess the prevalence of comorbidities in HIV/AIDS patients treated at a Special Care Dentistry Center and to determine their impact on oral health and on clinical management during dental treatment. Additionally, we evaluated the influence of gender, age, and the time elapsed since HIV infection diagnosis on the prevalence of comorbidities. The results will help us identify additional risks, plan personalized treatments, prevent complications, improve patient quality of life, promote coordination among healthcare professionals, educate patients about their conditions, and ensure safety during dental procedures.

## Methods

This cross-sectional observational study was conducted following the STROBE (STrengthening the Reporting of OBservational studies in Epidemiology) checklist and received approval from the Ethics Committee of the institution where it was carried out (protocol number: 1.664.741). Key elements of the STROBE checklist that we followed in our study include comprehensive reporting on participant eligibility criteria, data sources, measurement, and statistical methods, enhancing transparency and reproducibility.

All research participants read and signed the Informed Consent Form in accordance with the Declaration of Helsinki. All PLWHIV routinely seen at the Special Care Dentistry Center from August 2007 to August 2017 who were over 18 years of age were included in this study. Patients with cognitive impairment were excluded because the reliable reporting of medical history may be compromised.

Two researchers trained in oral medicine and PLWHIV approach conducted all the interviews and examinations. Demographic data, harmful habits (smoking, alcoholism, illicit drugs), self-reported and diagnosed comorbidities, use of cART, time of since HIV infection diagnosis, and probable mode of HIV transmission were compiled in a form developed for this study. In this specific form we have listed questions related to Review of Systems (ROS). The researchers recorded both negative and positive responses. Additionally, when available, we compiled the results of laboratory tests performed within 90 days prior to the assessment, including CD4+ T lymphocyte count, viral load (VL), complete blood count (CBC), total cholesterol, triglycerides, and fasting glucose. The values of laboratory tests were categorized according to reference values as altered, normal, and critical (when applicable). CD4+ T cell count was categorized as < 200 cells/mm^3^, 200 to 499 cells/mm^3^, and ≥ 500 cells/mm^3^. Viral load was classified as undetectable, < 10,000 copies/mm^3^, and ≥ 10,000 copies/mm^3^.

Data were analyzed using the Statistical Package for Social Science software (SPSS^®^ for Windows, version 20.0, SPSS Inc. Chicago, USA) and BioEstat^®^ version 5.3 (São Paulo, Brazil). Descriptive statistics of the data was performed. In the bivariate analysis, Pearson’s chi-square, linear association, and Fisher’s exact tests were used to determine the possible associations between comorbidities and demographic aspects and HIV infection. Variables with p≤0.20 were included in binary logistic regression models. They were selected based on their clinical relevance and statistical significance in bivariate analyses to identify and control for potential confounders.

Models were created for comorbidities associated with the presence of lipodystrophy, for PLWHIV over 50 years of age, male, infection time of more than 10 years and more than 20 years. The results were expressed as adjusted odds ratio and 95% confidence interval (95%CI), and associations that reached p<0.05 were retained in the model. In all analyses, the significance level α = 5% was considered.

## Results

Between August 2007 and August 2017, 340 PLWHIV received dental treatment at the Special Care Dentistry Center. Among them, 38 were excluded because they were younger than 18 years of age, 27 showed cognitive impairment, and 37 did not sign the Informed Consent Form. Among the remaining 238 participants, the mean age was 46 years, ranging from 22 to 70 years; 64.7% were male, and 73.1% were white. HIV transmission through heterosexual contact (50.0%) was most frequently reported, followed by male homosexual sex at 32.8%, and injectable drug use at 5.5%. One hundred and fifty-one participants (151/238; 63.4%) were under 50 years old at the time of data collection, while 87 (36.6%) were 50 years old or older. Ninety-five percent of the participants (226/238; 95%) were using cART at the time of data collection. Out of the total, 71 individuals (71/238; 29.8%) reported illicit drug use at some point, 59 (59/238; 24.8%) were active smokers, and 85 (85/238; 35.7%) had a history of alcoholism (Table 1).

**Table 1:** Baseline sociodemographic, clinical, and sexual behavior data of the people living with HIV (PLWHIV) included in this study.

Participants characteristics	Total n = 238 (100%)
Gender	
Male	154 (64.7)
Female	84 (35.3)
Age	Mean 46.18; Standard deviation 9.49
Ethnicity	
White	174 (73.1)
Black	64 (26.9)
HIV transmission mode	
Heterosexual	119 (50.0)
Homosexual	78 (32.8)
Injectable drug use	13 (5.5)
Multiple risk factors	11 (4.6)
Unknown	10 (4.2)
Blood transfusion	05 (2.1)
Vertical	02 (0.8)
Treatment	
cART*	221 (92.9)
Others	5 (2.1)
No therapy	12 (5.0)
Smoking	
Smokers	59 (24.8)
Former smoker	71 (29.8)
Non-smoker	108 (45.4)
Ilicit drugs	
Current use	15 (6.3)
Former use	56 (23.5)
Non- user	167 (70.2)
Alcohol	
Heavy drinkers	85 (35.8)
Former drinker	12 (5.0)

*Cart: combined antiretroviral therapy.

The vast majority of participants (229/238; 96%) in this study exhibited and/or reported at least one non-HIV-related comorbidity. The most prevalent comorbidities reported by participants were sexually transmitted infections (STIs) at 48.7% (116/238), followed by anemia at 38.7% (92/238), and pneumonia at 35.3% (84/238) (Table 2). The most frequent comorbidities presented by the participants at the time of the study were psychiatric disorders at 44.1% (105/238), followed by lipodystrophy at 40.8% (97/238), and hypertension at 26.5% (63/238) (Table 3).

**Table 2:** Comparison of self-reported medical history comorbidities in the studied population by age.

Self-reported comorbidities	Total	< 50 yrs.	≥ 50 yrs.	p-value
	(n = 238; 100%)	n = 151 (63.4%)	n = 87 (36.6%)	
STIs	116 (48.7)	72 (47.7)	44 (50.6)	0.768*
Anemia	92 (38.7)	64 (42.4)	28 (32.2)	0.156*
Pneumonia	84 (35.3)	51 (33.8)	30 (37.9)	0.613*
Herpes zoster	68 (28.6)	47 (31.1)	21 (24.1)	0.317*
Tuberculosis	58 (24.4)	39 (25.8)	19 (21.8)	0.594*
Toxoplasmosis	32 (13.4)	24 (15.9)	08 (9.2)	0.207*
Meningitis	20 (8.4)	13 (8.6)	07 (8.0)	0.880*
Cancer	17 (7.1)	12 (7.9)	05 (5.7)	0.709**

STIs: sexually transmitted infections; *Chi-square test; **Fischer’s Exact Test.

**Table 3:** Comparison of comorbidities at the time of the appointment in the studied population by age.

Comorbidities	Total	< 50 yrs.	≥ 50 yrs.	p-value
	(n = 238; 100%)	n = 151 (63.4%)	n = 87 (36.6%)	
Psychiatric disorders	105 (44.1)	71 (47.0)	34 (39.1)	0.293*
Lipodystrophy	97 (40.8)	54 (35.8)	43 (49.4)	0.041*
Hypertension	63 (26.5)	33 (21.9)	30 (34.5)	0.033*
Hepatitis C	45 (18.9)	30 (19.9)	15 (17.2)	0.744*
Diabetes mellitus	20 (8.4)	08 (5.3)	12 (13.8)	0.042*
Thyroid disorders	08 (3.4)	03 (2.0)	05 (5.7)	0.239**
Articular disorders	07 (2.9)	06 (4.0)	01 (1.1)	0.427**
Angina *pectoris*	06 (2.5)	02 (1.3)	04 (4.6)	0.262**
Mitral prolapse	05 (2.1)	05 (3.3)	00 (0.0)	0.213**
Heart murmur	05 (2.1)	03 (2.0)	02 (2.3)	0.872**
Recent acute myocardial infarction	04 (1.7)	02 (1.3)	02 (2.3)	0.968**

*Chi-square test; **Fischer’s Exact Test.

**Figure 1:**
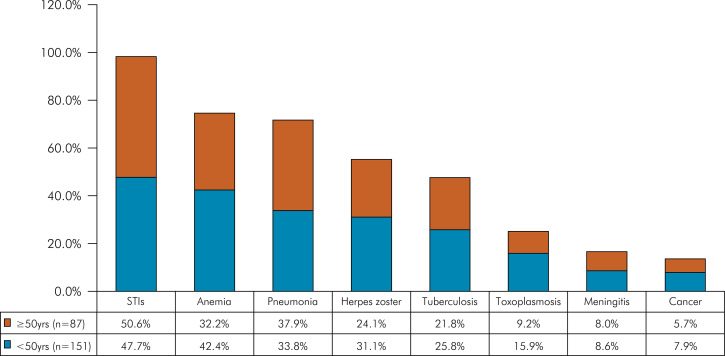
Self-reported medical history comorbidities after HIV infection in the studies population by age (< 50yrs. and ≥ 50 yrs.).

**Figure 2:**
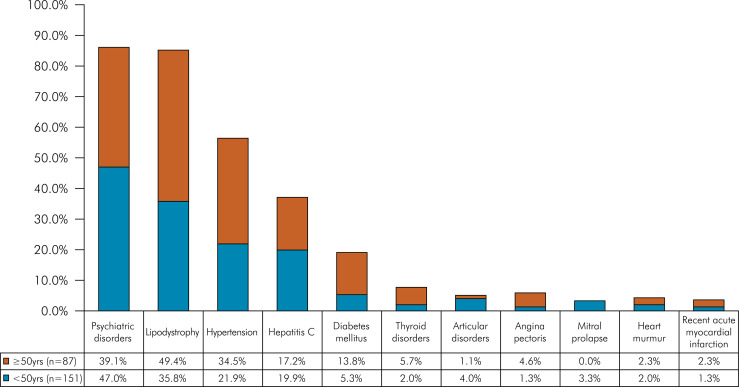
Comorbidities presented at the appointment in the studied population by age (< 50yrs. and ≥ 50 yrs.).

In the bivariate analysis, there was a higher proportion of males among PLWHIV reporting STIs, tuberculosis, and cancer, and a higher proportion of females reporting anemia (p < 0.05). Males were 4 times more likely to have STIs and 2.5 times more likely to have tuberculosis (p < 0.05), and they were 63% less likely to have anemia (p < 0.05).

In the group of PLWHIV aged ≥ 50 years there were more individuals with diabetes mellitus (12/87; 13.8%), STI (44/87; 50.6%), hypertension (30/87; 34.5%), and lipodystrophy (43/87; 49.4%) compared to the group of PLWHIV aged <50 years (p < 0.05). The chance of PLWHIV developing diabetes mellitus was 2.6 times greater in the group of individuals aged > 50 years (p < 0.05) (Table 3).

When considering the timing of HIV diagnosis, 85 individuals (85/238; 35.7%) were diagnosed within the past 10 years, whereas 153 (153/238; 64.3%) were diagnosed more than a decade ago. Hypertension (p = 0.046), diabetes mellitus (p = 0.013), lipodystrophy (p < 0.001), and hepatitis C (p = 0.015) were more frequently seen in the latter group.

When comparing the comorbidities of participants diagnosed with HIV 20 years ago or less (n = 198) to those diagnosed more than 20 years ago (n = 40), we observed a higher prevalence of thyroid disorders (p = 0.029), psychiatric disorders (p = 0.010), and lipodystrophy (p = 0.002) in the latter group, with long-term HIV/AIDS. The long-term HIV/AIDS group was three times more likely to experience lipodystrophy and twice as likely to develop psychiatric disorders (p < 0.05) (Table 4).

**Table 4:** Multivariate logistic regression for HIV-related comorbidities.

Comorbidities	OR adj (95%CI)	p-value
Lipodystrophy – Model 1		
Diabetes mellitus		
No	1	< 0.001
Yes	9.77 (2.77–34.38)	
Age (≥ 50 years) – Model 2		
Diabetes mellitus		
No	1	0.048
Yes	2.61 (1.01–6.75)	
Syphilis		
No	1	0.044
Yes	1.88 (1.02–3.47)	
Gender (male) – Model 3		
STIs		
No	1	<0.001
Yes	3.99 (2.19–7.28)	
Anemia		
No	1	0.002
Yes	0.37 (0.19–0.69)	
Tuberculosis		
No	1	0.016
Yes	2.55 (1.18–5.48)	
Time of HIV diagnosis (> 10 years) – Model 4		
Lipodystrophy		
No	1	<0.001
Yes	3.27 (1.78–6.01)	
Hepatitis C		
No	1	0.010
Yes	2.93 (1.29–6.64)	
Herpes Zoster		
No	1	0.028
Yes	2.12 (1.08–4.13)	
Time of HIV diagnosis HIV (> 20 years) – Model 5		
Lipodystrophy		
No	1	0.003
Yes	2.99 (1.44–6.19)	
Psychiatric disorders		
No	1	0.046
Yes	2.13 (1.01–4.47)	
Toxoplasmosis		
No	1	0.011
Yes	3.09 (1.29–7.35)	

STIs: sexually transmitted infection; OR adj: adjusted odds ratio; CI95%: 95% confidence interval. p = Wald Test probability.

Model 1 – variables included: diabetes mellitus, Omnibus Test = < 0.001. R2 Cox & Snell = 0.074. R2 Nagelkerke = 0.100.

Model 2 – variables included: lipodystrophy. anemia. systemic arterial hypertension. syphilis. diabetes mellitus. Omnibus Test = < 0.001. R2 Cox & Snell = 0.037. R2 Nagelkerke = 0.051. Hosmer and Lemeshow test = 0.865.

Model 3 – variables included: STIs. anemia. tuberculosis. syphilis. kidney disorders. meningitis. cancer. Omnibus Test = < 0.001. R2 Cox & Snell = 0.154. R2 Nagelkerke = 0.212. Hosmer and Lemeshow test = 0.697.

Model 4 – variables included: lipodystrophy. systemic arterial hypertension. syphilis. diabetes mellitus. hepatitis C. pneumonia. meningitis. herpes zoster. heart murmur. Omnibus Test = < 0.001. R2 Cox & Snell = 0.115. R2 Nagelkerke = 0.158. Hosmer and Lemeshow test = 0.592.

Model 5 – variables included: lipodystrophy. psychiatric disorder. thyroid disorder. toxoplasmosis. Omnibus Test = < 0.001. R2 Cox & Snell = 0.089. R2 Nagelkerke = 0.149. Hosmer and Lemeshow test = 0.686.

Values are n (%) unless otherwise stated. Bold values denote the statistically significant variables with p < 0.10, 95%CI. *STI comorbidities: hepatitis, human papillomavirus (HPV)/genital warts, gonorrhea and Chlamydia.

Of 225 patients for whom CD4 T+ values were available, 24 (10.7%) had a CD4 T+ count < 200 cells/mm^3^. The viral load (VL) was undetectable in 191 participants (84.1%; 191/227), while in 16 (7.1%; 16/227), the value was ≥ 10,000 copies/mm^3^. Seventeen participants showed low hemoglobin values, with two of them presenting critical values (Hb < 9 g/dL). Of 227 individuals, 17 had a neutrophil count lower than 1000/mm^3^ (Table 5).

**Table 5:** Laboratory values of the PLWHIV.

Variables	Total n (%)
CD4+ (cells/mm^3^)	225 (100)
< 200	24 (10.7)
200–499	57 (25.4)
≥ 500	144 (64)
Viral load (copies per mm)	227 (100)
Undetectable	191 (84.1)
< 10,000	20 (8.8)
≥ 10,000	16 (7.1)
Leukocytes (μL)	190 (100)
4,5–10 (normal)	164 (86.8)
< 4 (low)	25 (13.2)
Absolute neutrophil count (μL)	168 (100)
< 1,000 (altered)	17 (10.1)
≥ 1,000 (normal)	150 (89.9)
Hemoglobin (g/dL)	180 (100)
≥ 12 for women, ≥ for men (normal)	162 (90)
< 12 for women, <13 for men (altered)	15 (8.3)
< 9	2 (1.1)
Platelets (μL)	180 (100)
< 50,000	0
50–150,000	18 (10)
> 150,000	161 (90)
Glucose	129 (100)
< 100 mg/dL	26 (20.2)
≥ 100 mg/dL	103 (79.8)
Total cholesterol	140 (100)
≤ 200	91 (65)
> 200	49 (35)
Triglycerides	138 (100)
≤ 150	69 (50)
> 150	69 (50)

## Discussion

This observational cross-sectional study revealed differences related to age, gender, and time elapsed since HIV diagnosis in the burden of non-AIDS illnesses in HIV-positive individuals in Brazil.

The most reported comorbidities among the 278 interviewed HIV-positive patients were STIs (48.7%), pneumonia (35.3%), and herpes zoster (28.6%). At the time of consultation, the most commonly reported comorbidities were psychiatric disorders (44.1%), lipodystrophy (40.8%), and hypertension (26.5%).

Sexually transmitted infections were more predominant in male over 50 years of age, with an increased risk of presenting syphilis (28/87; 32.2%) (data not shown). The higher prevalence of STIs in male PLWHIV underscores the importance of screening and preventive education in dental settings. Certain behaviors as multiple partners, not using condoms, consuming alcohol and recreational drugs can increase the risk of contracting an STI. In addition, seniors have an active sexual life.^12^According to the Epidemiological Bulletin of the Brazilian Ministry of Health (2021), there was a slight decrease in HIV detection rates in people >60 years of age of both genders.^9^ However, a recent Brazilian study about sexual behavior among the elderly showed that about 30% of the participants reported having an active sexual life, with only 5.5% using condoms. These results reinforce that health professionals, including dentists, should expand their investigation of STIs based on the clinical history of patients and, particularly in the elderly, consider them as possibly sexually active and motivate them to take preventive measures. Dental professionals should be aware of the oral manifestations of STIs and provide appropriate referrals and education to manage and prevent these infections.^9,10^

In this study, 58 of the 238 participants (24.4%) reported occurrence of tuberculosis. A previous Brazilian study showed that the prevalence of tuberculosis in men with HIV in the largest city of the country, Sao Paulo, was 72.4%. These data point to the need for screening by the dentists, which can be performed by requesting complementary exams, as well as diagnosing possible oral manifestations of tuberculosis.^11^

Psychiatric disorders were highly prevalent in this study, and their incidence increased among PLWHIV with more than 20 years of infection. These results are similar to those of Brunneta et al. (2022), who found neuropsychiatric symptoms in 61% of the 2000 medical charts of HIV-positive adults on ART in Canada.^13^ Psychiatric disorders in PLWHIV are associated with a lower adherence to medication, increase in high-risk behaviors (unprotected sex, multiple sexual partners, and use of illicit drugs) and not seeking health services in general, including dental treatment.^13,14^PLWHIV are more prone to mood disorders, anxiety, and depression.^13^ Psychiatric disorders significantly impact oral health and dental treatment as patients in this situation may neglect oral hygiene, complicate the execution of operative procedures during dental visits, and fail to adhere to the dentist’s recommendations. Additionally, the dentist must be aware of reduced salivary flow related to certain medications and be cautious when prescribing medications due to the possibility of drug interactions. Dentists must be aware of signs of psychiatric disorders and refer the patient to psychiatry help if necessary. Psychiatric conditions can also deteriorate the patient’s global health and even affect the quality of oral health.

Lipodystrophy was seen mostly in PLWHIV over 50 years of age and with three times higher risk of occurring in individuals with HIV with more than 10 years since diagnosis. Over the last few decades, there has been a notable improvement in clinical outcomes of PLWHIV because of the availability of cART adherence. Nevertheless, this improvement has been associated with a rise in metabolic dysfunction, encompassing insulin resistance, dyslipidemia, and lipodystrophy. In certain studies, this shift in fat distribution has been observed in up to 80% of PLWHIV, mirroring additional metabolic changes attributed to HIV infection and prolonged use of cART.^15-17^ Protease inhibitors and non-nucleoside reverse transcriptase inhibitors are the drugs most associated with lipodystrophy. In addition to the aesthetic implications, PLWHIV with lipodystrophy suffer from stigmatization, low quality of life, and greater suicidal ideation. For this reason, they are more vulnerable to therapy withdrawal. The increased prevalence of lipodystrophy in older PLWHIV suggests the need for dental professionals to be vigilant about this condition, as it can affect facial aesthetics and complicate prosthodontic treatments. Early detection and management are crucial for providing appropriate cosmetic and functional dental solutions.^16,17^

The Brazilian public healthcare system provides complimentary services, including liposuction for the accumulation of fat in the dorsocervical region, abdominal and dorsal liposuction, breast reduction, surgical correction of gynecomastia, gluteal fat grafting, buttock augmentation with implants, facial fat grafting, and facial filling with polymethylmethacrylate.^16,17^ Dentists can perform the last two procedures, producing positive aesthetic results. The dentist should be mindful not only of the aesthetic implications associated with lipodystrophy but also of the potential metabolic imbalances linked to this condition.

The medical care for PLWHIV focuses on maintaining a good immunological status, reducing morbidity and mortality, and preventing HIV transmission. However, it is well known that the management of other comorbidities, whether HIV-related or not, is critical for PLWHIV to age well.^18^

People living with HIV over 50 years of age had a higher prevalence of diabetes mellitus and hypertension. It is possible that the pathogenesis of these two conditions is linked to aging, to HIV infection, and to cART use. The chance of PLWHIV developing diabetes mellitus in this study was 2.6 times higher in individuals >50 years. Thus, oral conditions related to hyperglycemia should be screened during the dental examination of PLWHIV including rapidly progressive periodontal disease, xerostomia, and candidiasis.^19^ It is also necessary for the dentist to know how to manage the main intraoperative complication in diabetic individuals, especially acute hypoglycemic crisis, which can be potentially harmful during dental care.^20,21^

A systematic review showed that the risk of hypertension was higher in individuals using cART than in cART-naïve individuals.^20^ Thus, when treating PLWHIV, dentists need to be aware of the possible development of oral manifestations commonly seen in hypertensive people, such as hyposalivation/xerostomia and drug-induced gingival enlargement.^21,22^ It is also important that hypertensive crises are promptly diagnosed and managed. The clinical management of hypertensive HIV patients should follow the same approach as in non-HIV hypertensive individuals.

Studies that investigated endocrine disorders in PLWHIV are scarce. A recent study evaluated people with diabetes mellitus with or without HIV, noted a higher prevalence of thyroid disorders (especially subclinical hypothyroidism) in PLWHIV who had diabetes mellitus.^23^ Oral manifestations of hypothyroidism are uncommon. When they occur, they may include rough skin, hoarse speech, pallor, relaxation of the deep tendon reflex, weight gain, and peripheral and eyelid edema. These manifestations can be challenging to detect.^24^

Hematological changes with the most significant impact on dental treatment were anemia and neutropenia. Critical anemia (Hb<9g/dL) was diagnosed in 2 cases among 180 blood tests evaluated, and neutropenia (<1000 cells/mm^3^) in 17 cases in a total of 168. Even though these situations are rare, it is advisable for the dentist to request a complete blood count of their HIV-positive patient from time to time, especially when invasive dental treatment is needed. Anemia can significantly impact oral health, manifesting as mucosal pallor, atrophic glossitis, and heightened susceptibility to infections. Critical values for anemia, such as hemoglobin levels below 9 g/dL, are particularly concerning, as they can lead to inadequate tissue oxygenation, delayed healing, and an increased risk of infections following dental surgeries. Dental practitioners should include anemia in their differential diagnosis when encountering these symptoms and collaborate with medical professionals for comprehensive management. Neutropenic patients may experience prolonged healing time and an increased risk of post-operative infections, requiring prophylactic measures.^25^

## Conclusion

This study provides important insights into the unique dental care needs of people living with HIV (PLWHIV) in the era following combined antiretroviral therapy. Psychiatric disorders and severe hematological alterations emerge as potential limitations to dental treatment at a certain point. By providing detailed recommendations for clinical practice, this study contributes to a growing body of evidence that supports the integration of specialized dental care into the overall healthcare management of PLWHIV. These contributions are crucial to developing
